# Retraction: Spectroscopic and molecular modeling studies of binding interaction between the new complex of yttrium and 1,10-phenanthroline derivatives with DNA and BSA

**DOI:** 10.3389/fchem.2024.1433667

**Published:** 2024-05-21

**Authors:** Frontiers Editorial Office

Following publication, concerns were raised regarding the integrity of the images in the published figures. The authors failed to provide the raw data or a satisfactory explanation during the investigation, which was conducted in accordance with Frontiers’ policies. Given the concerns about the validity of the data, and the lack of raw data, the editors no longer have confidence in the findings presented in the article.

This retraction was approved by the Chief Editors of Frontiers in Chemistry and the Chief Executive Editor of Frontiers. The authors have not agreed to this retraction.

